# Development of the Droplet Digital PCR to Detect the Teliospores of *Tilletia controversa* Kühn in the Soil With Greatly Enhanced Sensitivity

**DOI:** 10.3389/fmicb.2020.00004

**Published:** 2020-01-30

**Authors:** Jianjian Liu, Chao Li, Ghulam Muhae-Ud-Din, Taiguo Liu, Wanquan Chen, Jianmin Zhang, Li Gao

**Affiliations:** ^1^State Key Laboratory for Biology of Plant Diseases and Insect Pests, Institute of Plant Protection, Chinese Academy of Agricultural Sciences, Beijing, China; ^2^School of Agriculture, Yangtze University, Jingzhou, China

**Keywords:** *Tilletia controversa* Kühn, dwarf bunt, droplet digital PCR, soil born disease, detection

## Abstract

**Background and Aims:**

The dwarf bunt disease of wheat is caused by *Tilletia controversa* Kühn. This pathogen is primarily involved in the stunted growth of wheat and affects seed quality. Many countries in the world have therefore imposed quarantine bans to prevent the spread of *T. controversa*. Morphological observations are the main method of detecting teliospores in soil. However, this is a lengthy and laborious process; this method is thus unable to quickly meet the demand for detection of teliospores in the soil.

**Methods:**

We compared PCR, real-time PCR and droplet digital PCR (ddPCR) for the qualitative and quantitative measurement of the teliospores of *T. controversa* in soil.

**Results:**

We suggest the use of ddPCR for detection of the soil samples, which was demonstrated to have the most sensitive detection at 2.1 copies/μL. In contract, SYBR Green I real-time PCR could detect 7.97 copies/μL of *T. controversa* in soil, and this sensitivity was 100 times more sensitive than that of simple PCR.

**Conclusion:**

This study was the first report using ddPCR techniques to detect *T. controversa* teliospores in soil with greatly enhanced sensitivity.

## Introduction

Dwarf bunt is a disease of international concern and is caused by *Tilletia controversa* Kühn, a fungus belonging to the basidiomycetes class of fungi (Hoffmann, 1982; Trione, 1982). Young (1935) first differentiated *T. controversa* from regular *Tilletia*, and it is considered to be a quarantine pathogen in several countries to stop the import of wheat contaminated with *T. controversa*. The teliospores of *T. controversa* primarily accumulate in soil. These spores are resistant to adverse environmental conditions and are able to survive in soil for up to 10 years under favorable conditions (Zohary and Hopf, 1993). For quarantine and regulatory purposes, the levels of *T. controversa* in wheat grains and soil samples are quantified. However, there have been no previous reports on using molecular methods for the detection techniques of *T. controversa* teliospores in soil.

The conventional methods for isolating and quantifying the teliospores of *T. controversa* in soil are complex and time consuming (Durán and Fischer, 1961; Hess and Frione, 1986). Additionally, isolation of teliospores by this conventional method can sometimes fail and this could lead to a mistake in reporting the presence of teliospores in the fields. Therefore, it is essential to develop a fast and accurate molecular approach for detection and quantification of *T. controversa* teliospores in soil.

Currently, molecular techniques to differentiate *T. controversa* from other similar *Tilletia* species are mainly focused on genetic diversity (Pimentel et al., 1998; Pimentel, 2000), PCR (Kochanová et al., 2004), repetitive extragenic palindromic PCR (Rep-PCR) (McDonald et al., 2000; Zupunski et al., 2011), primer-mediated asymmetric-PCR (RM-PCR) together with SYBR Green I and Taqman real-time PCR was also constructed with the detection limit of 0.1 fg and 1.0 fg, respectively (Yuan et al., 2009). Pieczul et al. (2018) reported to identify *Tilletia* spp. with loop-mediated isothermal DNA amplification (LAMP), at detection limit of DNA concentration is 0.001 ng/μL, while which could not differentiate *Tilletia laevis, Tilletia caries*, and *T. controversa* separately. Multiplex PCR was applied to differentiate *T. controversa* from *T. caries* with detection limit of 10 fg (Nian et al., 2007). Moreover, Gao et al. (2010, 2011) developed Sequence Characterized Amplified Region (SCAR) primers marker for molecular identification of *T. controversa*. Real-Time PCR technology has been widely used as a rapid method to detect and quantify plant pathogens with a high degree of specificity, sensitivity, reliability and repeatability (López et al., 2003; Alemu, 2014; Fang and Ramasamy, 2015). Gao et al. (2014) have also used fluorescent dye and probes to establish a quantitative real-time fluorescence PCR detection system and through this method, they were able to lower the limit of detection to 0.1 fg/μL with increased sensitivity. However, these molecular detection methods are not sensitive enough to detect the teliospores of the pathogen in the soil directly.

Droplet digital PCR (ddPCR) has emerged as a sensitive technology which is capable of amplifying a highly diluted single-molecule in a droplet and then uses a fluorescent labeling probe to detect the target molecule (Hindson et al., 2011; Pinheiro et al., 2012). Moreover, ddPCR does not require external nucleic acid standards for absolute measurement of pathogen DNA. The ddPCR method distributes the sample into thousands of independent nanoscale droplets. Therefore, ddPCR eliminates issues with inhibition, reduces deviations of reaction factors in samples, is able to accurately identify target molecules from large amounts of non-target molecules and uses digital PCR and a Poisson distribution to calculate the original concentration of the target molecule in a sample (Vogelstein and Kinzler, 1999; Pohl and Iem, 2004; Morisset et al., 2013; Gutiérrez-Aguirre et al., 2015). The ddPCR method has been used for molecular identification, quantification and evolutionary analysis and provides better amplification efficiencies and trace nucleic acid detections (Hindson et al., 2011; Koch et al., 2016). Until now, there has been no studies using this technique for the detection of the teliospores in the soil. In this study, we compared ddPCR with PCR and real-time PCR for the detection and quantification of *T. controversa* in soil.

## Materials and Methods

### Sample Processing

Soil samples were collected (each sample for tested were taken from five points collected in the same test field and each point had three technical replicates) and filtered through a 40-mesh strainer. The fine soil was then weighed and packed into small sealed bags. These bags were labeled with sampling location, sample weight, and sampling time. The soil samples were stored at 4°C for 1 day for further processing and analysis.

### Extraction of Soil DNA and Synthesis of the Primers

Fast DNA^TM^ SPIN Kit for Soil (MP Biomedicals, Santa Ana, CA, United States) was used to extract total DNA from each soil sample according to the manufacturer’s instructions. Soil sample DNA (2 uL) was used for concentration detection by NanoDrop 3300 (Thermo Fisher Scientific, Waltham, MA, United States). The isolated DNA was stored at −80°C for later use (3 h later). The primers used for this study was previously reported (Gao et al., 2014; Table 1), which was based on the specific DNA fragment from *T. controversa*, and produces a 372 bp amplicon in PCR. The target sequence of real-time PCR and ddPCR is “ACGACCG ACTTTCCGAG AGCCTGCCTC TCC CTACCAT GGACCCCGGC TTCAAGAACG ACTTGCGGTC CCTCCACACG GATACCTCGG CCTTCTTGAT GCCTTC GTCC CACAC”. Primer synthesis were completed by Sangon Biological Engineering Technology and Services Co., Ltd. (Shanghai, China).

**TABLE 1:** The primers used for PCR, real-time PCR and ddPCR.

Primer sequences	Application	References
5′-TGGTGGTCGGGAAAGATTAGA-3′/ 5′-GGGACGAAGGCATCAAGAAG-3′	PCR	Gao et al., 2014
5′-ACGACCGACTTTCCGAGAGC-3′/ 5′-GTGTGGGACGAAGGCATCAA-3′	Real time-PCR and ddPCR	Gao et al., 2014

### Preparation and Dilution of Plasmid DNA Standard

A subsample of the total soil DNA was used as a template for PCR amplification. The total reaction system volume was 50 μL, including 25 μL of PremiSTAR HS (TaKaRa, Beijing, China), 1 μL of forward primer (10 μM), 1 μL of reverse primer (10 μM), 1 μL of template DNA (10 ng/μL), and 22 μL of ddH_2_O. PCR amplification conditions were as follows: initial denaturation at 94°C for 5 min (min), followed by 30 cycles of amplification with denaturation at 94°C for 30 s (s), annealing at 55°C for 30 s, and extension at 72°C for 30 s and then a final extension at 72°C for 10 min. Following completion of the PCR, total products were tested by agarose gel electrophoresis on a 1% agarose gel and the DNA bands were recovered from the agarose gel using Agarose Gel DNA Recovery Kitfrom (ComWin Biotech, Beijing, China).

TA cloning was then performed using the TA ligation cloning reaction system in a total volume of 10 μL including 5 μL of 2 × Solution buffer (ComWin Biotech, Beijing, China), 4 μL of the recovered DNA, 1 μL of T-vector (ComWin Biotech, Beijing, China). The ligation was completed at 22°C for 4 h. TA ligation cloning of recovered PCR products was carried out using DH5α competent cells (ComWin Biotech, Beijing, China). Colonies were picked and dissolved in 10 μL of sterile water and 1 μL of this solution was used as a template for colony PCR. The products of the colony PCR were sequenced through Sangon Biological Engineering Technology and Services Co., Ltd. (Shanghai, China). The EasyPure^®^ Plasmid Mini Prep Kit (TransGen Biotech, Beijing, China) was used to extract plasmid DNA for use as a standard in absolute quantification. This plasmid DNA standard (18 ng/μL) was serially diluted 10-fold (10^–1^ – 10^–9^) and 2 μL of each diluted solution was used as template to develop a standard curve.

### Detection of the Teliospores of *T. controversa* in Soil by PCR

The serially diluted plasmid DNA standards and the DNA from the soil samples were subjected to PCR in tandem. The total volume of the PCR amplification reaction was 25 μL including 12.5 μL of 2 × *Taq* PCR mix (Tiangen Biotech, Beijing, China), 1 μL of forward primer (10 μM), 1 μL of reverse primer (10 μM), 1 μL of template DNA (10 ng/μL), and 9.5 μL of ddH_2_O (Tiangen Biotech, Beijing, China). The PCR cycling conditions were: initial denaturation at 94°C for 5 min; 35 cycles of denaturation at 94°C for 20 s, annealing at 60°C for 20 s, and extension at 72°C for 30 s; following by a final extension step at 72°C for 7 min. The PCR products were stored at 4°C. Later, 10 μL of each PCR product was mixed with 2 μL 6 × Loading Buffer (Tiangen Biotech, Beijing, China) and ran on a 1% agarose gel for electrophoresis analysis. The teliospores of *T. laevis* are a very similar pathogen to *T. controversa*, so the DNA of *T. laevis* was used as a negative control (Russell and Mills, 1994). The test was repeated for three times.

### Detection the Teliospores of *T. controversa* in the Soil by Real-Time Fluorescence-Based Quantitative PCR

Real-time PCR was performed in a total reaction volume of 20 μL and included 10 μL of 2 × SYBR Green qPCR Mix (TransGen Biotech, Beijing, China), 0.5 μL of forward primer (10 μM), 0.5 μL of reverse primer (10 μM), 2 μL of template DNA (10 ng/μL), and 7 μL of nuclease-free water (TransGen Biotech, Beijing, China). The primers used in each of the PCR types were the same as mentioned in the previous sections. After the whole reaction system was well mixed and centrifuged, aliquots were loaded onto a 96-well PCR plate (0030128605, Eppendorf, Germany). Three biological and technical replicates of real-time PCR reaction were designed for each sample and 2 μL of nuclease-free water was used as a control on each plate. The ABI 7500 real-time PCR system (Applied Biosystems, Carlsbad, CA, United States) was used with the following reaction program settings: predenaturation at 95°C for 30 s, 40 cycles of denaturing at 95°C for 5 s, and annealing at 60°C for 40 s, followed by generation of melt curves using the following settings: 95°C for 1 min, 65°C for 1 min, and a single temperature rise of 0.5°C from 65°C to 95°C, holding at each temperature for 5 s. Real-time PCR raw data will be analyzed by ABI 7500 Software V2.3^1^. For the criteria for specific of the sample showed single clear peak in dissociation curve, for the efficiency will be calculated based on the slope of the calibration curve [10^ (−1/slope)−1] and for the evaluation of Tm, the curve should be single with high efficiency. The test was repeated for three times.

### Detection of the Teliospores of *T. controversa* in the Soil by Droplet Digital PCR (ddPCR)

A flowchart of the ddPCR method using whole process was showed in Supplementary Figure S1. The DNA was detected and quantified with QX100^TM^ Droplet Digital^TM^ (Bio-Rad, Pleasanton, CA, United States). The ddPCR reaction mix was composed of 10 μL of ddPCR Super mix for Probes, 1.8 μL of forward primer (10 μM), 1.8 μL of reverse primer (10 μM), 0.6 μL of probe (FAM 5′-ACGACTTGCGGTCCCTCCACA-3′ TAMRA), 2.0 μL of DNA template (10 ng/μL), and 3.8 μL of ddH_2_O. Droplets were prepared using droplet-generating cards (186-4007, BIO-RAD, Hercules, CA, United States) and a droplet generator (QX200, BIO-RAD, United States). PCR master mix (40 μL) and 70 μL of droplet-generating oil (186-3005, BIO-RAD, Hercules, CA, United States) were added to a droplet-generating card. The card was then capped with a special septum and placed into the droplet generator to generate droplets. Three biological and technical replicates were designed for each sample. The generated droplet emulsion was transferred to a new 96-well PCR plate (Eppendorf) and amplified in a C1000 Touch Thermal Cycler (Bio-Rad). The ddPCR was carried out in two steps with the following program settings: initial denaturing at 95°C for 10 min followed by 10 cycles of denaturing at 94°C for 15 s, annealing at 58°C for 30 s, and extension at 72°C for 30 s. This was followed by 30 cycles of denaturing at 94°C for 15 s, annealing at 60°C for 30 s, and extension at 72°C for 30 s. After the thermal cycling, the plates were transferred to a droplet reader (QX200, BIO-RAD, Hercules, CA, United States) and data acquisition were obtained through QuantaSoft (Version, 1.7.4, Bio-Rad, provided with the ddPCR system) analysis^2^ (Huggett et al., 2013). Single well thresholding was used to group droplets using the software’ s default internal algorithm. To provide a better estimate of the number of positive and negative droplets and to increase reproducibility of results, the Javascript program “dedinetherain”^3^ was used to set the threshold florescence amplitude. Positive controls used to calculate fluorescence thresholds consisted of runs using the sample containing *T. controversa* DNA. The experiment was repeated for three times.

## Results

### Detection of Teliospores of *Tilletia controversa* in Soil by PCR

**FIGURE 1:**
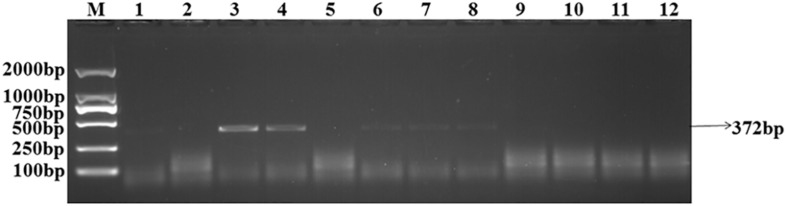
Amplification of the target DNA from soil samples with specific primers. *Line 1–10*, *T. controversa* soil samples; *line 11*, *Tilletia laevis* soil samples; *line 12*, ddH_2_O; *M*, DL2000 Marker (100, 250, 500, 750, 1,000, 2,000 bp); *black arrow*s show the target band of 372 bp.

**FIGURE 2:**
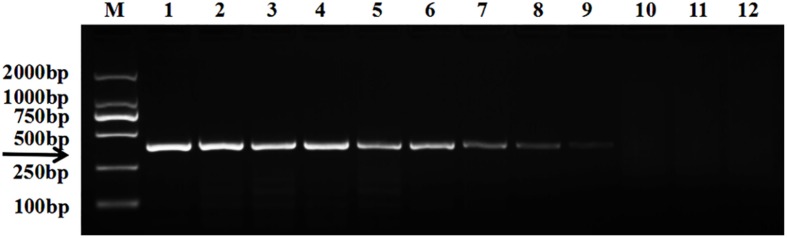
Electrophoresis tests of the plasmid DNA standard. *Line 1*, *T. controversa* teliospores DNA; *line 2–10*, the ten-fold serially dilutions of plasmid DNA standard (CN = 7.97 × 10^8^–7.97 × 10^0^); line *11, T. laevis* DNA; line *12*, ddH_2_O; *line M*, DL2000 Marker (100, 250, 500, 750, 1,000, 2,000 bp); *black arrow* shows the target bands of 372 bp.

The target bands were not well resolved while some samples had no band amplification at all (Figure 1). PCR tests showed that there were low levels of *T. controversa* teliospores in the soil samples which decreased PCR efficiency and due to this, PCR amplification products were affected. The test results of the plasmid DNA serially diluted standards showed that, like *T. controversa* teliospore DNA, each of seven serially diluted plasmid DNA standards (Copy Number (CN) = 7.97 × 10^8^ – 7.97 × 10^2^) could be obviously amplified into a 372 bp band (Figure 2). However, these bands were not detected in the last two serially diluted plasmid DNA standards.

### Detection of the Teliospores of *Tilletia controversa* in the Soil by Real-Time PCR

**FIGURE 3:**
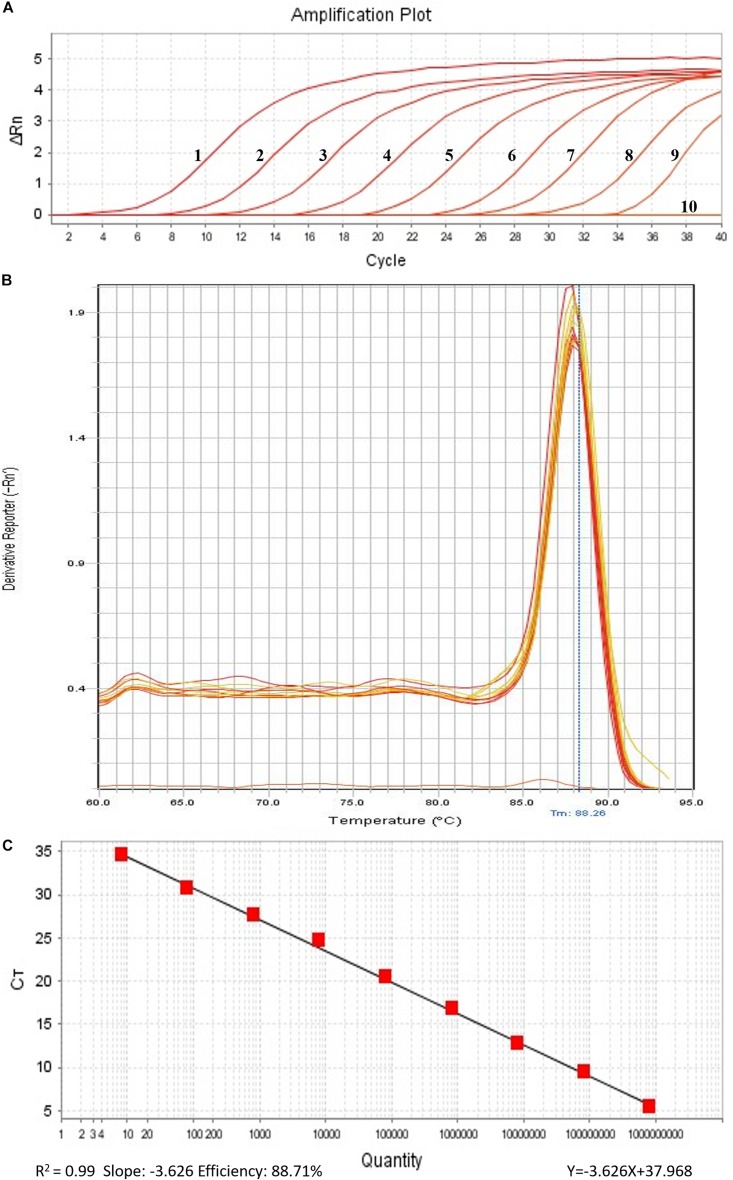
Establishment of standard curve by SYBR Green I Real-Time PCR. **(A)** Real-time amplified plot, *red lines 1–9*, ten-fold serially dilutions of plasmid DNA standard (CN = 7.97 × 10^8^–7.97); *line 10*, negative control ddH_2_O. **(B)** Melt curve of SYBR Green I (peak temperature at 88.26°C). **(C)** Standard curve.

**FIGURE 4:**
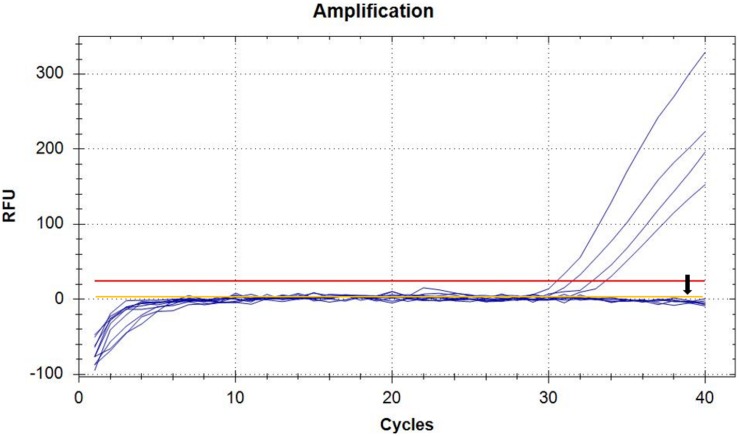
Detection of soil samples by SYBR Green I Real-Time PCR. Real-time amplified plot, *yellow line* with *black arrow* represents negative control *T. laevis*, and the *blue lines* represent the amplified curves of *T. controversa* soil samples. The red line represents threshold value.

In order to perform real-time PCR using SYBR Green I dye, we used a standard curve consisting of nine serial dilutions of plasmid DNA as templates (Figure 3). For plasmid DNA standards, real-time PCR showed a detection sensitivity of 0.018 fg (CN = 7.97) (Figure 3A) which was 100-fold more sensitive than the PCR approach (CN = 7.97 × 10^2^) (Figure 2). As demonstrated by the melt curve (Tm = 88.26°C) in Figure 3B, the amplification was highly specific and the primers showed good efficiency in amplification of the product. The standard curve had a high correlation coefficient (*R*^2^ = 0.99) and the PCR efficiency was 88.71% (Figure 3C). Theoretically, real-time PCR based on SYBR Green I is capable of detecting a trace amount of *T. controversa* teliospores in soil with a detection sensitivity two orders of magnitude higher than that of PCR. The same ten soil samples were selected and extracted DNA was used for real-time PCR detection with SYBR Green I. The results demonstrated that in contrast to simple PCR, real-time PCR was more sensitive and succeeded in detecting trace amounts of *T. controversa* in the soil samples. Additionally, this method was able to detect a corresponding number of copies in the soil samples (Figure 4). *Tilletia laevis*, which is very similar to *T. controversa*, was not detected, demonstrating that this method has a high degree of specificity and is able to distinguish between *T. controversa* and *T. laevis.* These results demonstrate that real-time PCR has a limit of detection of 7.97 copies/μL which is much lower than traditional PCR.

### Detection and Quantification of the Teliospores of *Tilletia controversa* in Soil by ddPCR

**FIGURE 5:**
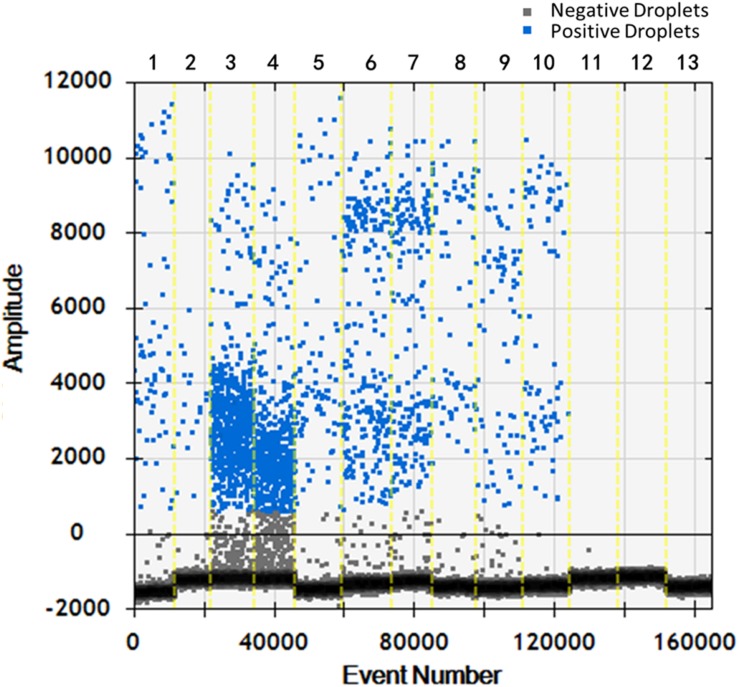
Distribution diagram of droplets of soil samples by droplet digital PCR (ddPCR) detection. *1–10*, *T. controversa* soil samples; *11–12*, *T. laevis* soil samples; *13*, ddH_2_O control; *blue bots* are positive droplets, and *black bots* are negative droplets.

**FIGURE 6:**
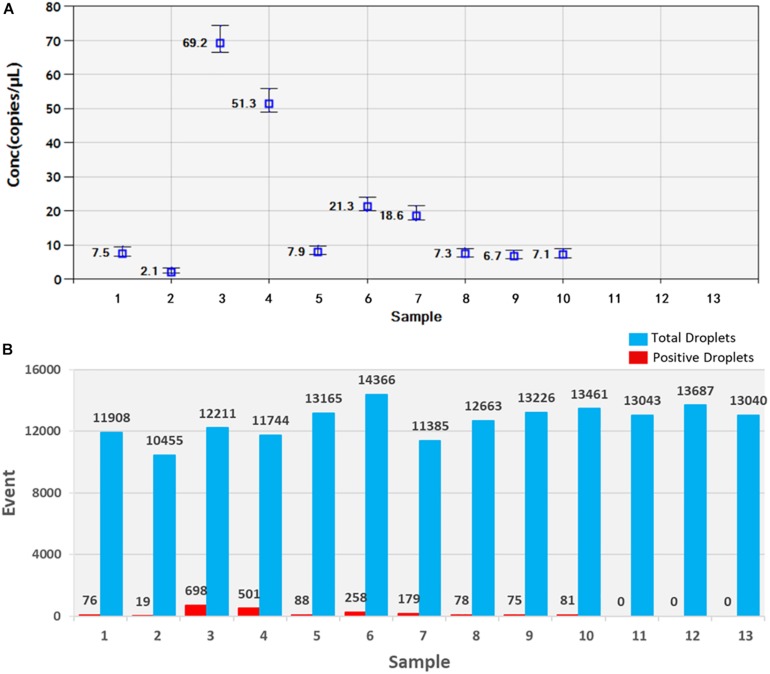
Statistic analysis of soil samples by ddPCR detection. **(A)** Positive copy number analysis, *1–10*, *T. controversa* soil samples; *11–12*, *T. laevis* soil samples; *13*, ddH_2_O control. **(B)** Number analysis of droplets, *1–10*, *T. controversa* soil samples; *11–12*, *T. laevis* soil samples; *13*, ddH_2_O control; red pillars are positive droplets, and *blue pillars* are total droplets (positive+negative).

Soil samples were chosen and DNA of *T. controversa* teliospores was extracted and detected using ddPCR. The results were further analyzed by using QuantaSoft. The threshold was “600” in this study (Supplementary Table S2) and which was set up automatically by the QuantaSoft software (Bio-Rad), by applying the signals observed in no templated controls together with on a sample with a target concentration within the linear range and with well-discriminated positive and negative droplets, which was close to the limit of detection of positive droplets, and combined the detection result of PCR and real-time PCR. For ddPCR, 10,000 droplets were used which is an accurate and reliable number. More blue-droplet points indicate the presence of an increased number of positive droplets in a sample and thus, greater copy numbers in the ddPCR product and higher concentrations of *T. controversa* in the sample. A zero-positive droplet is considered as indicating there was no detection of *T. controversa*. As a result, more direct test results can be obtained by using an area and distribution chart of blue point clusters as mentioned (Figure 5). Even though we found serials dilution of plasmid DNA standard could detect DNA of *T. controversa* with the limit detection of 0.9 copies/μL (Supplementary Figures S2, S3), while in this study, the lowest soil sample for tested by ddPCR was contained 2.1 copies/μL of *T. controversa* DNA (Figure 6A), the concentrated droplet fluorescence intensity was observed in most samples with a great number of droplets. In addition, there were not any positive droplets observed in samples that contained *T. laevis* (Figure 5). Statistical analysis of positive droplet quantities suggested that the ddPCR system was successful and effective for detection of *T. controversa* in soil (Figure 6A) and number analysis of droplets were showed in Figure 6B. Based all the above results, together with the criteria for ddPCR (Lievens et al., 2016), this method was capable of distinguishing *T. controversa* from *T. laevis*, and increased the lower limit of detection of DNA in soil to the 2.1 copies/μL. In comparison to PCR and real-time PCR, ddPCR had greater sensitivity and was able to obtain accurate measurements of positively expressed copies.

## Discussion

For soilborne diseases, identification and quantification of plant pathogens in soil is important for studying the degree of establishment of the disease in plants. Most fungal plant pathogens are characterized and quantified based on morphology following successful isolation from the soil (Beales, 2012). Recent advances in molecular identification and diagnosis have made PCR and real-time PCR-based detection very common for fungal pathogens such as *T. controversa* (Gao et al., 2010, 2011). In this study, qualitative and quantitative detection of *T. controversa* traces in soil was compared between three molecular methods of detection (PCR, real-time PCR, and ddPCR) with an aim to establish a high-sensitivity identification and quantification system by comparing the copies/μL from real-time PCR (Lee et al., 2006) and ddPCR. As demonstrated (Figure 6), traditional PCR was capable of detecting high concentrations of DNA from *T. controversa* teliospores but was not sensitive enough to detect trace amounts of *T. controversa* teliospores in soil. The limitations of this method make it prone to lower test accuracy and therefore, it is necessary to use a more sensitive molecular tool. In this experiment, SYBR Green I real-time PCR dye was used to detect trace *T. controversa* in soil samples. This approach specifically amplified *T. controversa* and no amplification of *T. laevis* was detected (Figure 4A). These results suggest that the real-time PCR method is 100-fold more sensitive than the simple PCR and was more sensitive than limits of detection reported (Yuan et al., 2009; Zouhar et al., 2010).

As we mentioned above, all of the experiments were repeated for three times, and the results were reproducible (Supplementary Table S1). The results obtained by ddPCR were better than those obtained by the other two kinds of methods. In this study, the ddPCR method detected 2.1 copies/μL of *T. controversa* DNA in soil samples (CN = 2.1, Figure 6A, Supplementary Table S1), demonstrating that accurate copy numbers could be obtained for all of the samples (Figure 6B). In addition, the closely related species, *T. laevis*, was not detected using ddPCR which suggests that the ddPCR method can accurately quantify trace levels of *T. controversa* in soil with enhanced specificity. In this study, the template was the total DNA from soil, the DNA was extracted by Fast DNA^TM^ SPIN Kit for Soil (MP Biomedicals, Santa Ana, CA, United States), the detection sensitivity of the teliospores in soil sample is dependent on the efficiency of the genomic DNA isolation kit which is used in this method. Therefore, the sensitivity of detection will vary according to DNA isolation kit. The positive droplets were in the sample of 1–10 with DNA of *T. controversa*, while totally not found in another three soil samples which containing the DNA of similar pathogen *T. laevis* and ddH_2_O. So, the experiment successfully differentiated the soil sample containing DNA of *T. controversa* from the soil containing DNA of *T. laevis*. To best of our knowledge, this is the first report on the use of ddPCR for the detection of *T. controversa* in soil and differentiate *T. controversa* from the soil sample which containing the teliospores of *T. laevis*.

Although high levels of dwarf bunt disease depend on low temperature and moisture conditions provided by deep and persistent snow cover (Tyler and Jensen, 1958), the spore population of *T. controversa* in soil is also a necessary condition for disease outbreak. Previous reports showed that only 8 teliospores per square centimeter in the soil can lead to the development of dwarf bunt by *T. controversa* (Goates and Peterson, 1999). Molecular detection of *T. controversa* by real-time PCR and ddPCR from the soil samples demonstrated that the copy number of *T. controversa* in soil samples from a dwarf bunt field were 5 ∼ 10 times higher than the *T. controversa* copy number of soil samples from normal field. Similarly, the lowest *T. controversa* content in soil samples from a dwarf bunt field was 96 mg (equivalent to 5 teliospores per gram soil), indicating a lower content of soil inoculum than previous studies had shown (8 teliospores per gram soil) (Goates and Peterson, 1999). Moreover, both detection systems of real-time PCR and ddPCR could be beneficial to the risk analysis of introducing dwarf bunt into other nations or areas by soil.

In recent years, comparison of ddPCR and real-time PCR has demonstrated that ddPCR has greater accuracy and reliability (Hindson et al., 2011; Pinheiro et al., 2012; Floren et al., 2014; Wiencke et al., 2014; Jernej et al., 2016). The results from our study support the use of ddPCR instead of PCR and real-time PCR as a more accurate and sensitive method to detect the teliospores of *T. controversa* in soil. This is also the first report using this technique to detect the teliospores of *T. controversa* in the soil.

## Data Availability Statement

All datasets generated for this study are included in the article/Supplementary Material

## Author Contributions

LG designed the experiment and wrote the manuscript. CL and JL did the experiment. GM, TL, WC, and JZ provided the materials and approved the manuscript.

## Conflict of Interest

The authors declare that the research was conducted in the absence of any commercial or financial relationships that could be construed as a potential conflict of interest.

## Footnotes

1 https://www.thermofisher.com/cn/zh/home.html

2 https://www.advanceduninstaller.com/QuantaSoft-736b5bf4ca629699d2ca4b0db11e8e05-application.htm

3 http://www.definetherain.org.uk

## Supplementary Material

FIGURE S1 A flowchart of the ddPCR method.

FIGURE S2 Distribution diagram of droplets of plasmid DNA standard by droplet digital PCR detection. 1–5, the ten-fold serially dilutions of plasmid DNA standard (CN = 7.97 × 10^2^–7.97 × 10^–2^); 6, *T. laevis* soil samples; 7, ddH_2_O control. blue bots are positive droplets, and black bots are negative droplets.

FIGURE S3 Statistic analysis of plasmid DNA standard by droplet digital PCR detection. **(A)** Positive copy number analysis, 1–5, the ten-fold serially dilutions of plasmid DNA standard (CN = 7.97 × 10^2^–7.97 × 10^–2^); 6, *T. laevis* soil samples; 7, ddH_2_O control. **(B)** number analysis of droplets, 1–5, the ten-fold serially dilutions of plasmid DNA standard (CN = 7.97 × 10^2^–7.97 × 10^–2^); 6, *T. laevis* soil samples; 7, ddH_2_O control; red pillars are positive droplets, and blue pillars are total droplets (positive+negative).

TABLE S1 Soil samples tested and results for the *Tilletia controversa* detection by three different methods.

TABLE S2 Raw data for ddPCR detection of soil samples.
