# Supplementary figures and images for: Development of the Droplet Digital PCR to Detect the Teliospores of Tilletia controversa Kühn in the Soil With Greatly Enhanced Sensitivity
Source: Front Microbiol. 2020 Jan 30;11:4. doi: 10.3389/fmicb.2020.00004 (PMC7002547; doi:10.3389/fmicb.2020.00004)

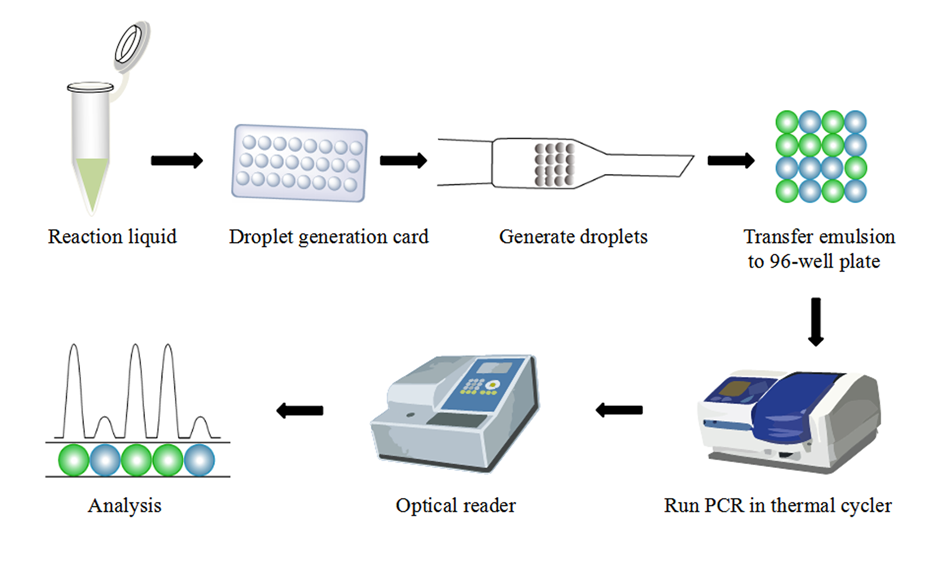

Supplement: FIGURE S1 — A flowchart of the ddPCR method. [file Image_1.TIF]

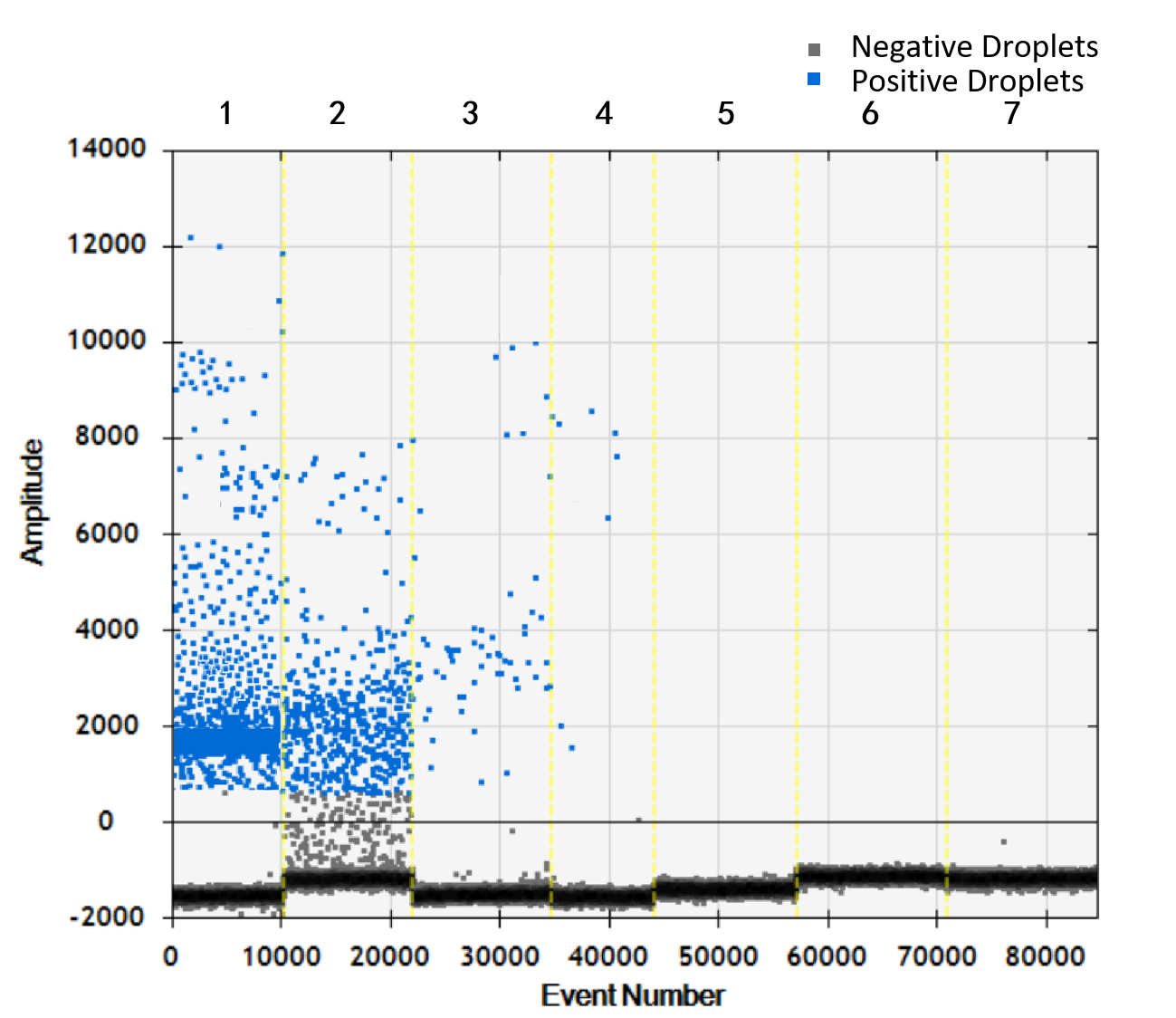

Supplement: FIGURE S2 — Distribution diagram of droplets of plasmid DNA standard by droplet digital PCR detection. 1–5, the ten-fold serially dilutions of plasmid DNA standard (CN = 7.97 × 10^2^–7.97 × 10–2); 6, T. laevis soil samples; 7, ddH2O control. blue bots are positive droplets, and black bots are negative droplets. [file Image_2.TIF]

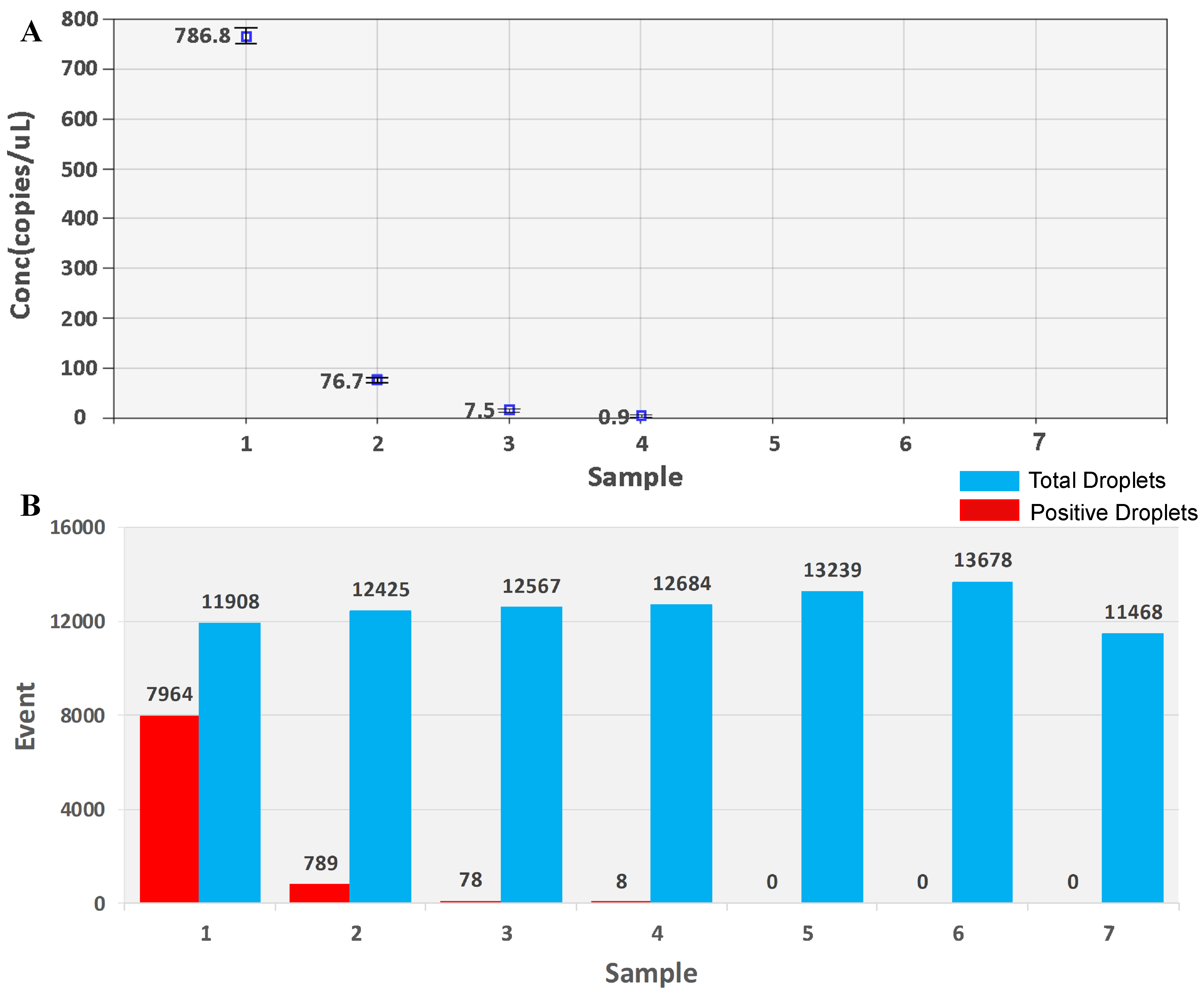

Supplement: FIGURE S3 — Statistic analysis of plasmid DNA standard by droplet digital PCR detection. (A) Positive copy number analysis, 1–5, the ten-fold serially dilutions of plasmid DNA standard (CN = 7.97 × 10^2^–7.97 × 10–2); 6, T. laevis soil samples; 7, ddH2O control. (B) number analysis of droplets, 1–5, the ten-fold serially dilutions of plasmid DNA standard (CN = 7.97 × 10^2^–7.97 × 10–2); 6, T. laevis soil samples; 7, ddH2O control; red pillars are positive droplets, and blue pillars are total droplets (positive+negative). [file Image_3.TIF]
