# Supplementary material for: Development of the Droplet Digital PCR to Detect the Teliospores of Tilletia controversa Kühn in the Soil With Greatly Enhanced Sensitivity
Source: Front Microbiol. 2020 Jan 30;11:4. doi: 10.3389/fmicb.2020.00004 (PMC7002547; doi:10.3389/fmicb.2020.00004)
Supplement: TABLE S1 — Soil samples tested and results for the Tilletia controversa detection by three different methods. [file Table_1.docx]

Supplemental Table 1 Soil samples tested and the results for the *T. controversa* detection by three different methods

| Soil samples | PCR detection | RT-PCR detection | ddPCR detection |
| --- | --- | --- | --- |
| 1 | undetected-372bp | No signal | 7.50±0.76 |
| 2 | undetected-no band | No signal | 2.10±0.18 |
| 3 | detected-372bp | 89.83±3.38 | 69.20±2.28 |
| 4 | detected-372bp | 59.42±2.04 | 51.30±1.89 |
| 5 | undetected-no band | No signal | 7.90±1.36 |
| 6 | detected-372bp | 26.37±3.18 | 21.30±1.09 |
| 7 | detected-372bp | 15.85±2.19 | 18.60±0.94 |
| 8 | detected-372bp | No signal | 9.30±0.68 |
| 9 | undetected-no band | No signal | 6.70±0.78 |
| 10 | undetected-np band | No signal | 7.10±0.86 |

Note: The reproducibility of the experiments was evaluated respectively, for PCR by running gels after PCR reaction three times, for RT-PCR and ddPCR were evaluated by the standard deviation based on the results of three times.
